# Pathophysiology, diagnosis, and herbal medicine-based therapeutic implication of rheumatoid arthritis: an overview

**DOI:** 10.1007/s10787-024-01445-8

**Published:** 2024-03-25

**Authors:** Charanjit Kaur, Yachana Mishra, Rajesh Kumar, Gurvinder Singh, Sukhraj Singh, Vijay Mishra, Murtaza M. Tambuwala

**Affiliations:** 1 School of Pharmaceutical Sciences, Lovely Professional University, Phagwara, 144411 Punjab India; 2 School of Bioengineering and Biosciences, Lovely Professional University, Phagwara, 144411 Punjab India; 3 Department of Food Civil Supply and Consumer Affairs, Amritsar, 143001 Punjab India; 4 Lincoln Medical School, University of Lincoln, Brayford Pool, Lincoln, LN6 7TS England, UK

**Keywords:** Rheumatoid arthritis, Bioimaging, Non-steroidal anti-inflammatory drug, Herbal medicine

## Abstract

Rheumatoid arthritis (RA) stands as an autoimmune disorder characterized by chronic joint inflammation, resulting in profound physiological alterations within the body. Affecting approximately 0.4–1.3% of the global population, this condition poses significant challenges as current therapeutic approaches primarily offer symptomatic relief, with the prospect of complete recovery remaining elusive. This review delves into the contemporary advancements in understanding the pathophysiology, diagnosis, and the therapeutic potential of herbal medicine in managing RA. Notably, early diagnosis during the initial stages emerges as the pivotal determinant for successful recovery post-treatment. Utilizing tools such as Magnetic Resonance Imaging (MRI), anti-citrullinated peptide antibody markers, and radiography proves crucial in pinpointing the diagnosis of RA with precision. Unveiling the intricate pathophysiological mechanisms of RA has paved the way for innovative therapeutic interventions, incorporating plant extracts and isolated phytoconstituents. In the realm of pharmacological therapy for RA, specific disease-modifying antirheumatic drugs have showcased commendable efficacy. However, this conventional approach is not without its drawbacks, as it is often associated with various side effects. The integration of methodological strategies, encompassing both pharmacological and plant-based herbal therapies, presents a promising avenue for achieving substantive recovery. This integrated approach not only addresses the symptoms but also strives to tackle the underlying causes of RA, fostering a more comprehensive and sustainable path towards healing.

**Graphic abstract:**

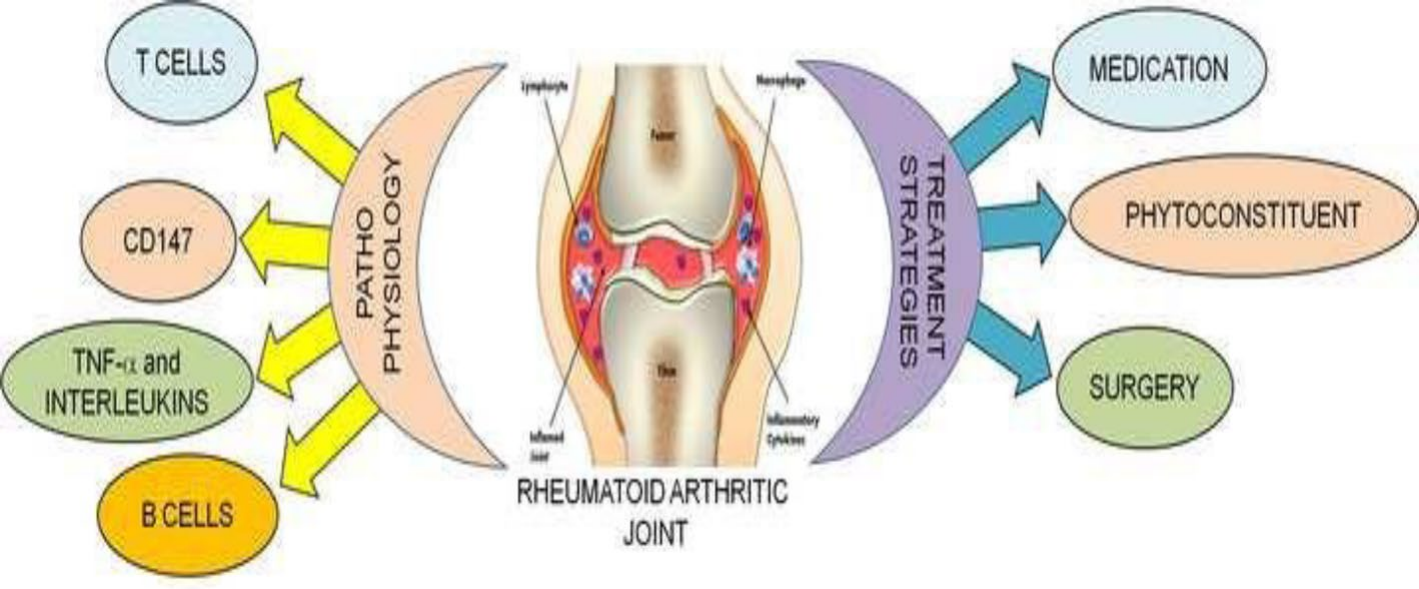

## Introduction

Rheumatoid arthritis (RA) is a chronic, inflammatory, systemic, autoimmune disease affecting approximately 0.4–1.3% of the world population with unknown etiology. In RA, synovitis, structural and joint bone damages followed by pain and disability are the cascade of immune reactions produced by both genetic and environmental factors (Ahmed et al. 2019). It initiates with inflammation of joint and synovial tissue proliferation, followed by massive cell infiltration and surplus formation of inflammatory mediators, finally resulting in cartilage and bone devastation (Baker et al. 2022) RA is also termed as “immortal cancer,” causing huge burdens on patients and society due to its higher incidence of RA(Cabrales-Rico et al. 2017; Chen et al. 2017; Littlejohn and Monrad 2018). Over the last 30 years, there has been no permanent cure for the patients suffering from RA as these only reduce pain and inflammation, decelerate the disease progression and prevent joint damage (Barnabe 2020; Conigliaro et al. 2019). The commonly used drugs for RA consist of non-steroidal anti-inflammatory drugs (NSAIDs), disease-modifying antirheumatic drugs (DMARDs), corticosteroids, biologics, and their combinations (Zhao et al. 2021a, b). These therapeutic agents do not provide persistent diminution but may cause immunity suppression, leading to severe complications. In case of infections, pathogenic cells are eliminated by antigen-specific therapies, utilizing existing autoantigens like heat shock proteins, and their derived peptides, leaving the immune system of the body unaffected (Cabrales-Rico et al. 2017). Due to the side effects linked with these therapeutic agents, including hepatotoxicity, cardiovascular complications, and gastrointestinal lesions, 30–50% of patients do not respond effectively to the standard therapies, thereby decreasing the inclusive benefits of the treatment on a long-term basis.

Herbal products have been the source of many bioactive compounds with therapeutic potential, of which many eventually have been developed into drugs that are consumed worldwide for diverse disorders, including inflammatory and autoimmune diseases. Furthermore, a variety of herbal products belonging to the traditional systems of medicine are either already being used by patients with autoimmune diseases, including RA, with or without the primary physician’s knowledge, or are under investigation for their therapeutic potential (Venkatesha et al. 2016). Thus, the utilization of phytoconstituents which are effective in relieving the inflammation and thereby alleviate the chances of occurrence of side effects associated with allopathic drugs, could be a potential alternative such as sinomenine, total glucosides of paeony, and triptolide (Doss et al. 2016; Han et al. 2016; Jia et al. 2016; Li et al. 2017; Voon et al. 2017). Thus, there is an urgent requirement to quest for more effective, safe, and economical drugs for relieving pain in joints and improving the quality of life of RA patients.

Natural plant products belonging to the traditional systems of medicine, such as traditional Chinese medicine and Indian Ayurvedic medicine, offer a vast and promising resource in this regard. With the number of RA patients increasing yearly, medicinal researchers are actively looking for cheap and effective alternative drugs with fewer side effects to treat RA. Recently, herbal medicines have been given more and more attention for their remarkable curative effects and fewer side effects (Luo et al. 2019; Newman and Cragg 2016). Herbal medicines have been used for the clinical management of RA for thousands of years, and their efficacy and safety have been proved by their long-term clinical application (Pu et al. 2016). Herbal medicines can act in multiple pathways to prevent and treat RA through numerous components, including flavonoids, alkaloids, phenylpropanins, terpenes, etc. The main pharmacological effects are related to pain relief, improvement of inflammation, immune function regulation, cartilage protection, reduction of pannus formation, inhibition of synovial hyperplasia, etc. (Zhang et al. 2019).

## Methodology

The qualitative systematic review analyzed globally accepted databases, including indexed and peer-reviewed journals from PubMed, Scopus, Medline, Google Scholar, and Research Gate. Papers from between 2001 and 2023 were included. The search was made using keywords such as rheumatoid arthritis, pathogenesis of rheumatoid arthritis, therapy of rheumatoid arthritis, herbal medicine for rheumatoid arthritis. The botanical names and families of the plants used for RA were mentioned after verification from published literature and databases. Data selection criteria are in accordance to botanical features, phytoconstituents, in vitro and in vivo models used, and clinical studies of RA. The inclusion criteria of the studies/reports in the present review are (i) plants reported for anti-arthritic activity, (ii) plants used in traditional systems and various polyherbal preparations, (iii) plants native to India and other areas such as America, Africa or Europe, and (iv) plants use under different models such as Complete Freud’s Adjuvant (CFA) and monosodium iodoacetate (MIA)-induced RA rat models.

### Etiology of rheumatoid arthritis

**Fig. 1:**
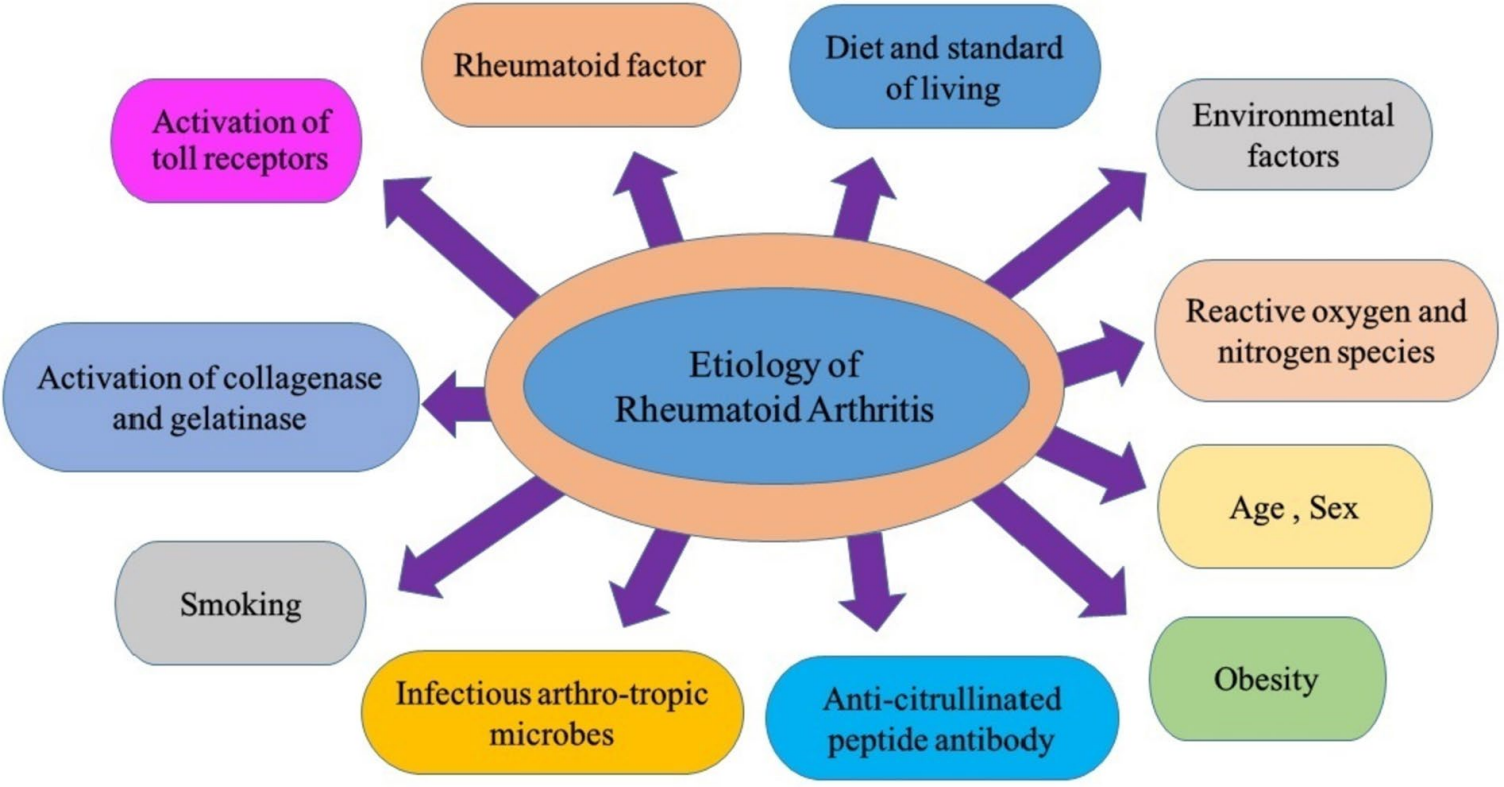
Etiology of rheumatoid arthritis

The etiology behind RA is still not precisely defined; however, specific research findings have provided evidence in favor of the involvement of various factors, as shown in Fig. 1 (Alam et al. 2017; Littlejohn and Monrad 2018; Daikh 2022).

### Pathophysiology of rheumatoid arthritis

**Fig. 2:**
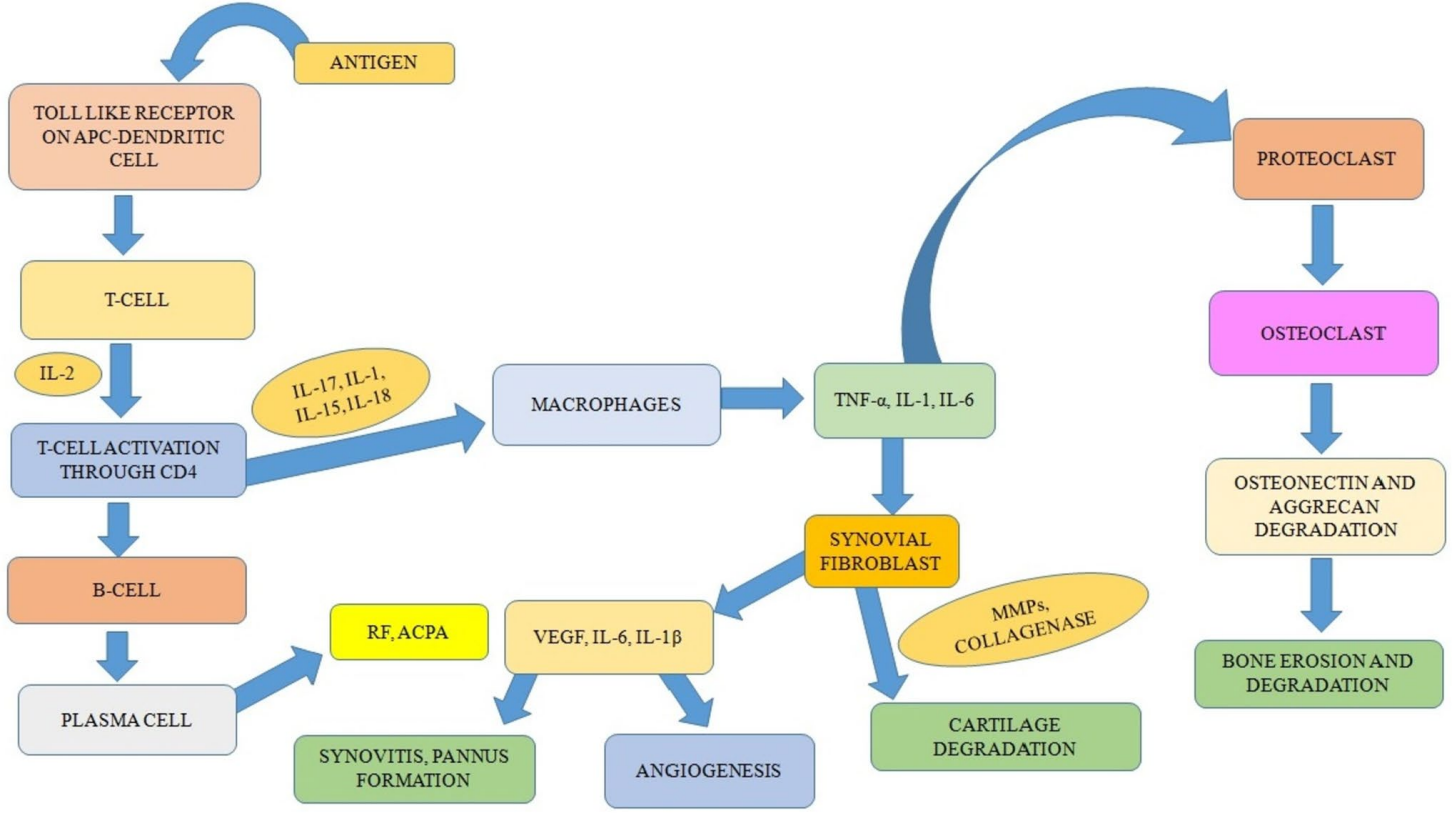
Pathogenesis of rheumatoid arthritis

In the pathogenesis of RA (Fig. 2), T cells, CD147, interleukins (ILs), and tumor necrosis factor-alpha (TNF-α) play crucial roles (Christman and Gu 2020). It has been described as follows:

### Function of T cells

Various reports advocated the connection of T cells in the pathogenesis of RA as it has been found in synovial tissue and fluids. There exists a relationship between specific major histocompatibility complex (MHC) class II alleles, an abundance of T cells, and this disorder (Alam et al. 2017; Melet et al. 2013). The helper T cell type 17 (Th17), through the production and discharge of TNF-α, IL-17F, IL-17, IL-22, and IL-21, has been considered as a cell adaptive immune response in RA (Quesniaux et al. 2013). T cells are triggered and produced due to the release of several cytokines (IL-32, IL-6, IL-15, IL-12, IL-18), co-stimulatory, and HLA class II molecules. The release of these intermediates has been linked to the abundance of myeloid cells such as macrophages, monocytes, basophils, neutrophils, erythrocytes, eosinophils, megakaryocytes or platelets, dendritic cells, and plasmacytoid dendritic cells in the synovium (Lebre et al. 2008), validating the use of abatacept, which blocks the interaction between T cells and dendritic cells or B cells through CD28 on the T cells with CD86 or CD80 on B cell or the dendritic cell, i.e., co-stimulatory signal. Autoreactive T cells have also been found against citrullinated self-proteins (Cantaert et al. 2009). The dendritic cells and macrophages are responsible for producing cytokines such as TGF-β, IL-6, IL-1b, IL-23, and IL-21, which provide an environment for differentiation of Th17 and suppress the differentiation of regulatory T cells (Genovese et al. 2014). In RA patients, the functional ability of regulatory T cells decreases, which is regulated by Foxp3 (forkhead box P3, master regulator) and TNF-α. In antigen nonspecific T-cell mediated activation, there is activation of macrophages and fibroblast through the interaction of ICAM-1 & LFA-1, CD200 & CD200L, and CD40 & CD40L (Noack and Miossec 2014).

### Role of CD147

The CD147 (EMMPRIN-an extracellular matrix metalloproteinase inducer) is a member of the immunoglobulin super family. CD147 induces matrix metalloproteinases (MMPs) expression. In the synovial membrane of patients suffering from RA, it has been found that cellular response to a molecular stimulus increases due to a rise in the number of receptors of CD147 on peripheral blood CD14þ monocytes. Reports have suggested that in the RA patients, CD147 expression on monocytes/ macrophages increases, and could be responsible for the enhanced secretion of MMP, cyclophilin A-mediated migration of the cell into the joints of the patient, and cell invasion, which is ultimately responsible for bone and cartilage destruction in the patients. The monoclonal antibody CD147/HAb18 reduces inflammatory cytokines, inhibits MMPs, and alleviates synovitis as well as cartilage erosion in immuno-deficient mice engrafted with human rheumatoid synovium tissue and cartilage. All these outcomes advised that CD147 can be a novel target for the treatment of RA (Guo et al. 2019).

### Role of TNF-α and interleukins

The TNF-α has a very vital function in RA in controlling the inflammatory response. There is an elevated TNF-α level in the synovium, blood, and synovial fluid of RA patients. The TNF-α acts both on fibroblast-like synoviocytes (FLS) and macrophages, facilitating the release of MMPs that enable the destruction of the tissue. The MMPs are involved in both pathologic destructions of the tissue and physiologic connective tissue remodeling. All the important structural proteins in the cartilage matrix are degraded by at least 19 human MMPs extracellularly (Huang et al. 2013; Roeleveld and Koenders 2015). The FLS is an important class of cells and chief contributor of the hyperplasia of synovium, which is involved in the pathogenesis and progression of RA by regulating the secretion of inflammatory mediators like TNF-α, IL-6, IL-1α, chemo-attractant protein-1 (MCP-1), IL-17, monocytes, cyclooxygenase-2 (COX-2), and IL-1β-inducible nitric oxide synthase (iNOS). It has been suggested that intracellular signaling molecules such as nuclear factor кB (NF-кB) and activator protein-1 (AP-1) are very activated in RA-FLS and contribute to the pathogenesis of RA. In basic cellular processes, like proliferation, apoptosis, differentiation, and immunity, NF-кB, and AP-1 are the chief transcription factors regulating gene expression.

TNF-α, NF-кB, IL-1b, AP-1, and Mitogen-activated protein kinases (MAPKs) intermediates get activated after receiving the pro-inflammatory stimuli and release pro-inflammatory chemokines, MMPs, inflammatory enzymes, adhesion molecules, and angiogenic factors resulting in neo-vascularization, arthritic joint destruction and chronic inflammation. Furthermore, it has been revealed during several pre-clinical and clinical trials that RA-FLS plays a significant role in RA through the manufacturing of receptor activator of NF-кB ligand (RANKL), which results in bone erosion through enhanced stimulation of osteoclast formation (Alam et al. 2017; Cantaert et al. 2009; Chen et al. 2017; Doss et al. 2016; Ganesan et al. 2016; Gao et al. 2017; Genovese et al. 2014; Guo et al. 2019; Huang et al. 2013; Lebre et al. 2008; McInnes and Schett 2017; Melet et al. 2013; Noack and Miossec 2014; Quesniaux et al. 2013; Roeleveld and Koenders 2015).

### Role of mast cells

Mast cells and macrophages act by releasing interleukins (IL-1, IL-6, IL-12, IL-15, IL-18, IL-23), TNF-α, reactive nitrogen and oxygen species, phagocytosis and antigen presentation, production of matrix-degrading enzymes and prostanoids (Shichita et al. 2012) and cause synovitis in RA through interaction with the Toll-like receptors (TLR), suppression of tumorigenicity 2 (ST2), Fc receptor ϵ and γ through innate immune system activation (Hueber et al. 2010; Németh and Mócsai 2012; Nigrovic and Shin 2015).

### Role of B cells

Due to the occurrence of different factors like proliferation-inducing ligand (APRIL), chemokines and B-lymphocyte stimulator (B LyS), synovial B cells get localized as T cell–B cell aggregates or tertiary lymphoid follicles in synovium in RA (Ohata et al. 2005). The pathogenic involvement of CD20^+^ B cells has been verified by rituximab used in patients suffering from RA. Moreover, clinical observations show that B cells are engaged in the RA pathogenesis beyond autoantibody production. The cytokine production and autoantigen, e.g., lymphotoxin-b or TNF-c, IL-6, and TNF-α, contribute to adaptive humoral immunity. Plant-derived natural products offer a vital and promising resource for new therapeutic agents for RA and other autoimmune diseases. Practitioners of the traditional systems of medicine prefer to use herbal extracts, either singly or in a formulation using multiple herbs. The principal mechanisms of RA prevention and symptom alleviation are the reduction of inflammatory mediators and inhibition of specific signaling pathways involved in the inflammatory response in RA.

### Diagnostic techniques of rheumatoid arthritis

The RA can be diagnosed by two methods, i.e., Clinical diagnostic methods and Radiographic diagnostic techniques, as discussed in Tables 1 and 2, respectively (Ebel and O’Dell 2021; Alghuweri et al. 2012; Deodhar 2015; Gotthardt et al. 2010; Haville and Deane 2022; Karnati et al. 2013; Kashyap et al. 2015; Kim et al. 2014; Llopis et al. 2017; Maharaj and Daikh 2022; Patil et al. 2011; Rezaei et al. 2013; Rosa Neto et al. 2009; Suter et al. 2011; Verma et al. 2002; Wakefield et al. 2005).

**Table 1:** Clinical diagnostic marker/parameter of RA

Markers/parameters involved	Observations/effect	References
Anti-cyclic citrullinated peptide antibody (ACPA)	Early detection of RA	Alghuweri et al. (2012)
Rheumatoid factor (RF)	Found in 80% of pre-articular phase of RA	Alghuweri et al. (2012)
Plasma proteins like fibrinogen	Affect ESR in RA patients	Verma et al. (2002)
Tumor necrosis factor-α (TNF-α)	Synovitis and leukocyte aggregation in RA	Kashyap et al. (2015)
C-reactive protein (CRP)	Systemic inflammation in RA	Kashyap et al. (2015), Rosa Neto et al. (2009)

**Table 2:** Radiographic diagnostic techniques

Technique	Observation/Effect	References
X-ray imaging: Digital dual-energy X-ray absorptiometry; X-ray radiogrammetry	Assess the anatomical changes, measure the bone mineral density, pre-articular bone resorption, joint space narrowing, periarticular bone erosions and irregular joint space	Llopis et al. (2017)
Ultrasonography	Detect degradation of small joints, the damaged synovium, cartilage tissues, inflammatory fluid, the swelling rate, imaging of the tendon sheaths and vascularization of synovial membrane	Wakefield et al. (2005)
Magnetic resonance imaging (MRI)	Edema of bone marrow and synovial inflammation during the advancement of RA at the indistinguishable state of inflammatory arthritis	Suter et al. (2011)
Bone scintigraphy	Swelling of multiple joints and tenderness	Kim et al. (2014)
Fluoro-2-deoxyglucose (^18^F-FDG) and technetium methylene diphosphonate (^99m^Tc MDP)	Probes used for the tracing of bone inflammation	Gotthardt et al. (2010)

### Therapy of rheumatoid arthritis

The treatment for RA is a mixed avenue of allopathic and non-allopathic strategies. Allopathic medications and surgical interventions are the most popular treatments for RA. Currently available drugs either used alone or in combination for the treatment of RA include Glucocorticoids (GCs), NSAIDs, and DMARDs of both biological origin (such as TNF Inhibitors, IL-1, IL-6, and B cell depleting drugs) as well as synthetic (conventional like Methotrexate (MTX) and targeted like JAK-Inhibitors) (Akram et al. 2021; Zago et al. 2020; Burmester and Pope 2017). Phytoconstituents are also taken by large populations (Swathi et al. 2021). Alternative therapies like physiotherapy, acupuncture, yoga, and massage are also popular among RA patients.

#### Medication

**Fig. 3:**
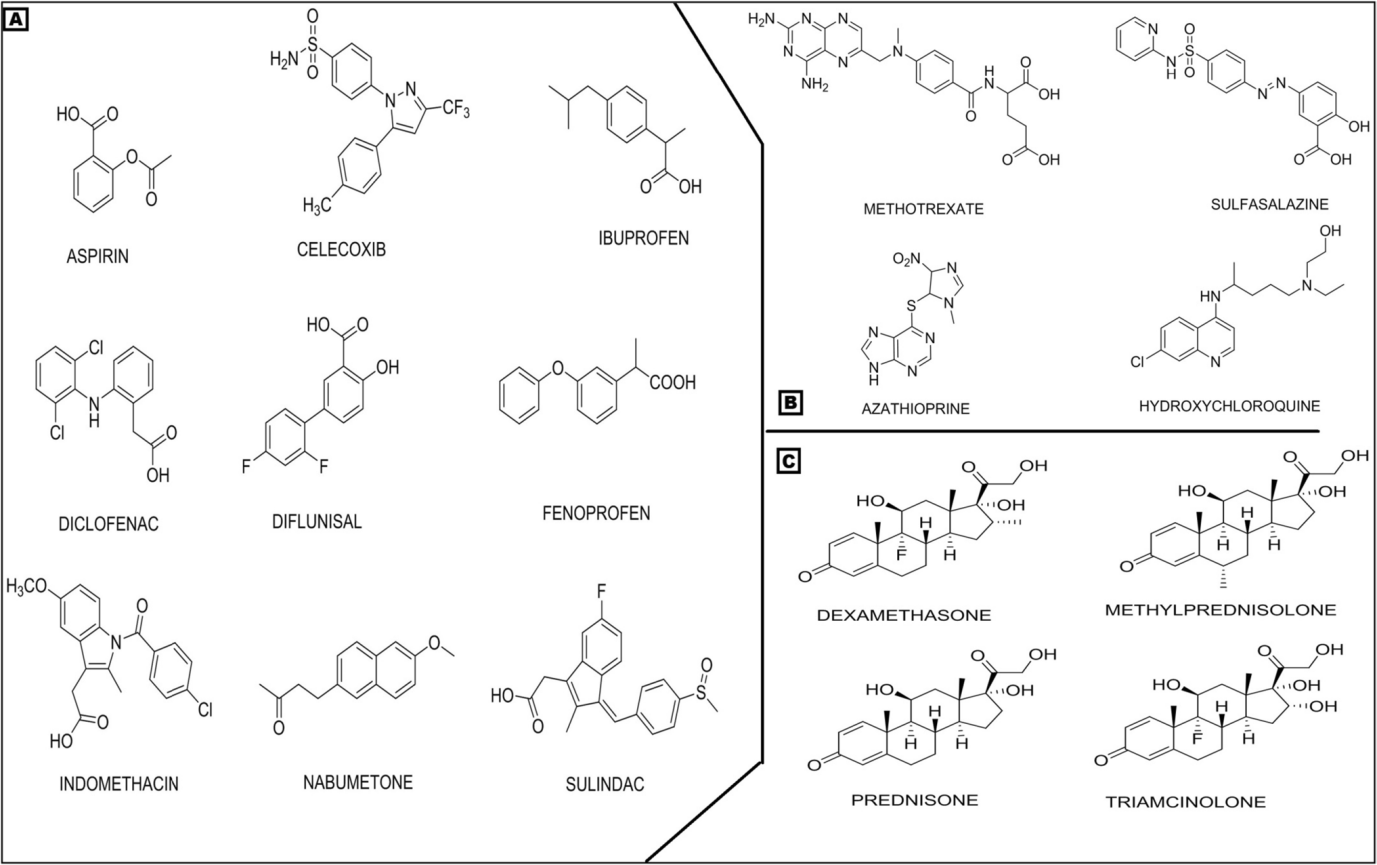
Some important classes of medicine: **A** NSAIDs, **B** DMARDs, and **C** corticosteroids used in rheumatoid arthritis

Synthetic drugs like corticosteroids, DMARDs, and NSAIDs reduce swelling, pain, and associated symptoms of RA (Fig. 3) (Zhao et al. 2021a, b; Wysham et al 2022). These drugs can either be used alone or in combination to diminish inflammation caused due to the activation of RA mediators.

#### Non-steroidal anti-inflammatory drugs

These drugs are COX enzyme inhibitors, which decrease pain and inflammation. The COX occurs in two natural forms, i.e., COX-1 and COX-2. During the arachidonic acid pathway, the COX or prostaglandin synthase produces prostaglandin E2 (PGE2), which is accountable for synovium inflammation and articular degradation of cartilage. NSAIDs inhibit COX-2 and COX-1 and, decrease synovial inflammation in RA (Stańczyk and Kowalski 2001). The NSAIDs are preliminary drugs, usually prescribed in patients without RA risk factors like multiple swollen joints, RF, mobility loss, and bone erosion. For better recovery, these can be given in combination with a low dose of glucocorticoids (Ebel and O’Dell 2021). However, some side effects such as renal malfunction, gastrointestinal disturbance, and increased cardiovascular risk are associated with NSAIDs. Some of the important NSAIDs used clinically are shown in Fig. 3A.

#### Disease modifying antirheumatic drugs

Based on their characteristic mechanism in RA, these drugs are classified as biological DMARDs and standard synthetic DMARDs. The action of synthetic DMARDs is intracellular (Kiely and Nikiphorou 2018). The first-line standard drug used in RA is MTX and about 40% of the RA patients are sensitive to it (Peres et al. 2015). Some of the important synthetic DMARDs are shown in Fig. 3B. Synthetic DMARDs such as MTX, Hydroxychloroquine (HCQ), Sulfasalazine (SSZ), Clofazimine (CLO), Leflunomide (LEF), Actarit, and Azathioprine show various side effects including myelosuppression, hepatic cirrhosis, interstitial pneumonitis, and gastrointestinal disorders (Zhao et al. 2021a, b).

Adalimumab and infliximab are the most commonly used biological DMARDs, which mimic the effects of natural biological agents generated by the immune system of the body by acting as TNF-α inhibitors (Barrios et al. 2017; Rein and Mueller 2017; Schoellnast et al. 2006). These frequently target the CD molecules of antigen-presenting cells, soluble cytokines, and B cells. The mechanism of action of biological DMARDs involves antagonism of TNF-α, IL-1 or IL-6 receptor. These agents reduce the formation of rheumatoid factors and antibodies, downregulate T cells activation and deplete B cells. Although these agents are effective, their usage is limited due to various associated side effects like hypersensitivity, renal disease, hypertension, cardiac complications, gastrointestinal damage, respiratory difficulty, ulcer, and immune suppression, leading to opportunistic infections. Some of the important biological agents useful in RA are mentioned in Table 3 (Alten et al. 2011; Bredemeier et al. 2014; Emery et al. 2009; Flouri et al. 2014; von Kempis et al. 2012; Koike et al. 2007; Kurrasch et al. 2013; Lee et al. 2014a, b; Nam et al. 2014; Schiff et al. 2014; Smolen et al. 2015; Takeuchi et al. 2016; van Vollenhoven et al. 2015; Weinblatt et al. 2014; Deane et al. 2023).

**Table 3:** Biological disease-modifying antirheumatic drugs

Biological agent	Target	Reference
Abatacept	T-cell co-stimulation blocker	von Kempis et al. (2012), Schiff et al. (2014)
Adalimumab	TNF blocker	Nam et al. (2014)
Anakinra	IL-1 receptor blockade	Nam et al. (2014)
Certolizumab pegol	TNF blocker	Smolen et al. (2015)
Etanercept	TNF blocker	Koike et al. (2007)
Golimumab	TNF blocker	Emery et al. (2009)
Infliximab	TNF blocker	Flouri et al. (2014)
Rituximab	B cell depletion	Bredemeier et al. (2014)
Biological agents in clinical trials		
Canakinumab and Sirukumab	Human anti-IL1ß monoclonal antibody	Alten et al. (2011)
Tofacitinib	Orally administered JAK inhibitor that selectively inhibits Janus kinase (JAK-1, JAK-2 and JAK-3)	Lee et al. (2014a, b)
Ofatumumab	Human anti-CD20 monoclonal antibody	Kurrasch et al. (2013)
Atacicept	Inhibits B cell maturation by blocking the binding BLyS & APRIL	van Vollenhoven et al. (2015)
Denosumab	Anti-RANK ligand antibody	Takeuchi et al. (2016)
Fostamatinib	Spleen tyrosine kinase (Syk) inhibitors; The Syk signaling pathway has an important role in the TNF alpha-induced expression of inflammatory cytokines	Weinblatt et al. (2014)

#### Corticosteroids

When glucocorticoid secretion from the adrenal gland is insufficient, stress and inflammation intervene. Corticosteroids, the analogs of cortisol, are used for anti-inflammatory and immune-regulatory functions. These constitute second-line therapy. These are prescribed for symptomatic relief as they reduce the inflammatory intermediates in the body like prostaglandins and leukotrienes to reduce inflammation. The mechanism of action of corticosteroids involves the prevention of phospholipid release, anti-inflammation, and immune suppression. Glucocorticoids like Dexamethasone, Betamethasone, Methylprednisolone acetate, Prednisolone phosphate, and Budesonide show many side effects such as hyperadrenocorticism, insulin resistance, atherosclerosis, hypertension, osteonecrosis and osteoporosis, obesity, and inhibition of wound repair. These are usually combined with other synthetic drugs when they are ineffective for RA separately (Gupta and Singh 2014). Corticosteroids used in the RA therapy are displayed in Fig. 3C.

#### Phytoconstituents

Before developing synthetic drugs, mankind was reliant on medicinal plants to get relief from a specific disorder. Regardless of the accessibility of numerous synthetic drugs for rheumatoid joint pain, the side effects restrict the utilization of the remedial treatment, and the likelihood of use of medicinal plants for the treatment of rheumatoid joint inflammation has expanded. A considerable number of plants have potential anti-arthritic constituents, and a few formulations are likewise endorsed by professionals in place of synthetic drugs (Kapoor et al. 2017). Engineered remedial medications act by one of the accompanying systems, i.e., a) modulating a specific cell, b) augmenting inflammatory mediator, and c) inhibiting tissue debasing enzymes. Yet, restoration of the immune system of the patient is not achieved. Such disadvantages can be overcome by using natural phytoconstituents, which are considered superior to allopathic medications and surgical treatments in various terms. Phytoconstituents are non-harmful and multifocal on the malady pathway. The rundown of some anti-arthritic plants, their phytoconstituents are portrayed in Table 4 (Ahmed 2010; Ali et al. 2007; Anilkumar 2010; Bang et al. 2009; Gupta and Singh 2014; Haleagrahara et al. 2017; Lee et al. 2013; Patil et al. 2011; Stańczyk and Kowalski 2001; Sun et al. 2006; Voon et al. 2017; Zaka et al. 2017; Zhou et al. 2017).

**Table 4:** List of some anti-arthritic plants, phytoconstituents, and their description

Phytoconstituent	Plant source	Description	References
Sitoindoside	*Withania somnifera*	Used in inflammation and arthritis	Gupta and Singh (2014), Sun et al. (2006)
Withaferin A and withanolide D	*W. somnifera*	Alkaloid used in arthritis	Ali et al. (2007)
Bartogenic acid	*Barringtonia racemosa*	Used in inflammation and arthritis	Patil et al. (2011), Ali et al. (2007), Haleagrahara et al. (2017)
Quercetin	*Cardiospermum halicacabum*	Used in inflammation and arthritis	Anilkumar (2010)
β-Boswellic acid	*Boswellia serrata*	Used in inflammation and arthritis; Reduced breakdown of glycosamino glycans	Bang et al. (2009)
Piperine	*Piper nigrum*	Natural alkaloid having activity in arthritis	Zhou et al. (2017)
Sinomenine	*Sinomenium acutum*	Used in rheumatoid arthritis	Ahmed (2010)
Cardamonin	*Boesenbergia rotunda*	Used in inflammation and arthritis	Voon et al. (2017)
Epigallocatechin	*Camellia sinensis*	Polyphenol used in arthritis	Lee et al. (2013)
Mangostin	*Garcinia mangostana*	Natural xanthonoid used in inflammation and arthritis	Zaka et al. (2017)

#### Bartogenic acid

Bartogenic acid (BA) isolated from *Barringtonia racemosa* was evaluated in arthritic rats in which arthritis was induced by Complete Freud’s Adjuvant (CFA). The results indicated that BA exhibited a protective role in rats against the haematological perturbations, body weight changes as well as primary and secondary arthritic lesions induced by CFA (doses 2, 5, and 10 mg kg^−1^ day^−1^, p.o.) with remarkable reduction in the rheumatoid factor and C-reactive protein (CRP), which are distinct serum markers of arthritis and inflammation. This result establishes the potency of BA in arthritis. However, further clinical investigations are mandatory to demonstrate the efficiency of BA in the treatment of several immuno-inflammatory ailments (Ali et al. 2007; Patil et al. 2011; Sun et al. 2006).

#### Quercetin

Quercetin (QU), a natural flavonoid, is found in hundreds of herbs, vegetables, and fruits. QU has numerous pharmacological potentials, such as anti-inflammation, anti-platelet aggregation, expanding coronary arteries, antitumor, lowering blood pressure, anti-allergy, etc. For a single oral dose of 4 g or 500 mg twice a day, no side effects were reported after continuous administration for a month (Haleagrahara et al. 2017). When analyzed on adjuvant-induced arthritis in mice, it exhibited anti-inflammatory action by lowering the production of macrophage inflammatory mediators by regulating NF-κB activity, and finally resulted in the inhibition of inflammatory cytokine expression and inflammatory reactions. QU can inhibit the activity of MMP-2, thereby inhibiting the degradation of the basement membrane and indirectly inhibiting angiogenesis and cartilage damage (Haleagrahara et al. 2017; Ji et al. 2013).

Askari and colleagues studied the effect of flavonoid QU (500 mg/day) supplementation in healthy male non-professional athletes for 2 months, with regular exercise. The results showed a remarkable lowering in the levels of CRP (Askari et al. 2012). QU having the ability to inhibit xanthine oxidase prevented the uric acid accumulation, which benefited the gout patients (Anand David et al. 2016).

In a pathological condition, QU did not show any significant alteration in CRP levels in women with RA (Javadi et al. 2017). The QU dosage continuously for 8 weeks significantly reduced after-activity pain, morning pain, and EMS (*p* < 0.05). In comparison to the placebo, hs-plasma TNF-α level, Health Assessment Questionnaire (HAQ) scores, DAS-28, and the number of patients were decreased in the QU group with active disease (*p* < 0.05). Thus, there were notified improvements in clinical symptoms, hs-TNFα, disease activity, and HAQ after 500 mg/day QU dose intake in women suffering from RA for 8 weeks (Javadi et al. 2017).

#### Epigallocatechin-3-gallate

Epigallocatechin-3-gallate (EGCG) is abundant in green tea and can be an effective adjunct therapy for the treatment. It has been reported to inhibit inflammatory mediators like PGE2, COX-2, NF-κB, iNOS, interferon-γ, TNF-α in arthritic joints collagen type II-induced arthritis (CIA) in pre-clinical studies (Ahmed 2010). Experiments conducted by Ahmed and colleagues have shown that pre-treatment of human chondrocytes with EGCG significantly prohibited the activities and expression of MMP-13 and MMP-1 (IC_50_ values 16.5 and 27 mM, respectively) in vitro in a dose-dependent manner. The EGCG was found to be effective in inhibiting IL-1b-induced MMP-1, MMP-3, and MMP-13 in human tendon fibroblasts (Ahmed et al. 2005; Ahmed 2010).

The EGCG suppresses pro-inflammatory cytokines and chemokines induced by TLR9 agonists in prostate cancer cells (Mukherjee et al. 2014). The anti-inflammatory and anti-cancer properties of EGCG are mediated by folate cycle disruption, adenosine release, and NF-kB suppression (Navarro-Perán et al. 2008). The EGCG of green tea suppresses autoimmune arthritis through Indoleamine-2,3-dioxygenase expressing dendritic cells and the nuclear factor, Erythroid 2 of the antioxidant pathway (Min et al. 2015).

#### Boswellic acid

Boswellic acid (BA) (*Boswellia serrata*, Burseraceae), pentacyclic terpene, is found in plants in various derivatives such as acetyl-11-keto-BA and 11-keto-BA. BA and its derivatives are therapeutically used in multiple ailments such as ulcerative colitis, cancer, hepatitis, inflammation, pain, cough, bacterial infection, and osteoarthritis. In pre-clinical evaluation, BA was reported to reduce cartilage loss, synovitis, and osteophyte formation and hence has a beneficial role in osteoarthritis and other joint disorder.

Clinical investigation of *B. serrata* extract provided statistically substantial improvement in patients suffering osteoarthritis and was well-tolerated with minor gastric disturbance. The anti-arthritic activity of BA is due to the inhibition of NF-ĸB, COX-2, LOX-5 (Anilkumar 2010).

The BA inhibited microsomal prostaglandin E synthase1 (mPGES-1), a terminal enzyme of PGE2 biosynthesis (IC_50_ = 3–30 μM) in IL-1beta-stimulated human A549 cells. Some other triterpene acids (i.e., tircuallic, lupeolic, and roburic acids) isolated from frankincense were also found to suppress mPGES-1 with increased potencies. 3α-Acetoxy-7,24-dienetirucallic acid and 3α-acetoxy-8,24-dienetirucallic acid inhibited mPGES-1 activity (IC_50_ = 0.4 μM, each). Docking simulations and structure–activity relationship (SAR) studies established structure-related interactions with mPGES-1 and its co-substrate glutathione (Verhoff et al. 2014). Acyl derivatives of BA also act as inhibitors of NF-κB and STATs (signal transducer and activator of transcription protein) determined against human HL60 cells after 48 h by MTT assay (Kumar et al. 2012).

#### Mangostin

Lee and colleagues have shown that mangostin extract possesses immunomodulatory activity due to the presence of α-, β- and γ-mangostin in the extract. These inhibited the secretion of TNF-α (IC_50_ = 2–11 μg/mL), and IL-6 in mice (ED_50_ < 100 mg/kg). It reduced the arthritis score in the CIA rats and the paw oedema in the carrageenan-induced rats (Lee et al. 2013).

#### Piperine

Bang and colleagues conducted the study on piperine and reported that it inhibited the expression of MMP-13 and IL-6 in a dose-dependent manner and reduced the production of PGE2 (concentrations 10–100 μg/mL). Piperine suppressed the migration of activator protein-1 (AP-1) into the nucleus in IL1β-treated synoviocytes. On days 4 and 8, piperine significantly reduced arthritic and nociceptive symptoms in rats. Histological staining revealed the anti-inflammatory effect of piperine especially in the ankle joints (Bang et al. 2009).

#### Sinomenine

The studies conducted by Zhou and colleagues and Feng and colleagues showed that the treatment with Sinomenine (SIN) decreased the occurrence and severity of anti-CII IgG and the antigen-specific splenocyte proliferation and carrageenan-induced arthritis. It was observed during the study that secretion of cytokines such as IL-5, IFN-γ, and generation of all isotypes of antibodies including anti-CII IgG1, IgE and IgG2a were found to be suppressed along with increased TGF-β secretion and with no effect on IL-10 production. These inferences proposed that anti-arthritic activity can be possible due to the suppression of Th1 and Th2 immune responses (Feng et al. 2007; Zhou et al. 2017). The anti-inflammatory effects of sinomenine may occur via the inhibition of pro-inflammatory cytokine and COX-2 production through the inhibition of MAPKs and NF-kB pathway activation by phorbol 12-myristate-13-acetate (PMA) along with A23187 stimulation in HMC-1 cells (human mast cells) (Oh et al. 2012).

#### Cardamonin

*Boesenbergia rotunda* (L) Mansf is the natural source of cardamonin (2′,4′-dihydroxy-6′-methoxychalcone). The study conducted by Voon and colleagues showed that cardamonin inhibited the release of the pro-inflammatory cytokines such as TNF-α, IL-6, and IL-1β, in vitro. This study was conducted in RA-induced rat model to determine the anti-arthritic properties of cardamonin, specifically on the pain and inflammatory response of RA. The anti-arthritic activity was evaluated using four different doses of cardamonin (5.0, 2.5, 1.25, and 0.625 mg/kg), through responses to the CFA-induced paw oedema, thermal hyperalgesia, and mechanical allodynia. Histological, behavioral, and biochemical studies in rats demonstrated that cardamonin exhibited substantial inhibition of RA-induced pain, inflammation, and advancement of joint destruction. In cardamonin-treated RA rats, results from ELISA showed significant inhibition in IL-6, IL-1β, and TNF-α levels in plasma. Overall, cardamonin showed noteworthy anti-arthritic potential in CFA-induced RA rat model (Voon et al. 2017).

In another study, the anti-inflammatory property of cardamonin was examined in IFN-γ/LPS-stimulated microglial cells. In this investigation, cardamonin demonstrated promising anti-inflammatory activity in microglial cell line BV2 by inhibiting the secretion of pro-inflammatory mediators, including TNF-α, PGE2, IL-6, IL-1β, and nitric oxide (NO). The inhibition of NO and PGE2 by cardamonin has resulted from the reduced expression of iNOS and COX-2, respectively (Chow et al. 2012).

#### Withanolide

Zaka and colleagues showed that anti-arthritic actions of withanolides can be because of the inhibition of stimulation of NF-kB and gene expression regulated by NF-kB (Zaka et al. 2017). Rasool and Varalakshmi examined *W. somnifera* root powder in MSU crystal-induced arthritis in rats. A substantial increase in paw volume and the levels of lysosomal enzymes in serum were observed in rats with arthritis, due to enhanced lactate dehydrogenase and β-glucuronidase levels in MSU incubated polymorpho-nuclear leucocytes. The levels of lactate dehydrogenase and β-glucuronidase reverted to near normal after treatment (500 to 1000 mg/kg). Thus, these have shown potent analgesic and antipyretic effects at different doses, without any sign of gastric damage. These inferences provide substantial proof for the suppressive action of withanolides present in *W. somnifera* root powder on arthritis through reduction of propagation of the inflammatory response, without producing any gastric damage (Rasool and Varalakshmi 2007).

#### Withaferin A

Sultana and colleagues targeted the synovial macrophages by injecting steroidal lactone withaferin A containing mannosylated liposomes (ML-WA) into adjuvant-induced arthritic (AIA) rats to develop an improved therapeutic method to treat RA through internalization of ML-WA in the primarily isolated synovial macrophages. It was observed that targeting the synovial macrophages via ML-WA reduced the severity of inflammation of the paw oedema, bone resorption, and oxidative stress (ROS and NO) in AIA rats through repolarization of M1 to M2 macrophage (Sultana et al. 2017; Zaka et al. 2017). The clinical evidence for bee venom acupuncture (BVA) for RA has also been reported (Lee et al. 2014a, b).

To check the safety and efficacy of traditional Chinese medicine Paeoniflorin (PAE) plus cervus and cucumis polypeptide injection (CCPI), a double-blinded study was conducted using control leflunomide (LEF) and MTX. Patients were randomly assigned to one of the three groups: PAE + CCPI, MTX + LEF, and MTX + LEF + CCPI. The CCPI groups responded better to the ACR20 during early treatment. After 6 months, ACR20 was similar in the three treatment groups. The maximum improvement in the two DMARD groups was higher than that in the PAE + CCPI group (*p* < 0.01). CCPI decreased the onset action of the DMARD therapy 4.6 times. The PAE + CCPI had significantly lower adverse event incidences than two DMARD groups. Thus, PAE + CCPI was observed to be an acceptable alternative to DMARDs and can also be used in adjunct with DMARDs to increase the rate of onset of action, when necessitated. Although not as effective as DMARDs, PAE can be a safer option to substitute DMARDs for long-term RA treatment in case of DMARD toxicity (Chen et al. 2013). Withaferin A down-regulated lipopolysaccharide (LPS)-induced COX-2 expression and PGE2 production by inhibiting STAT1/3 activation in microglial cells (Kyoung-Jin et al. 2011). In another study, Withaferin A inhibited iNOS expression and NO production by Akt inactivation and down-regulation of LPS-induced activity of NF-kB in RAW 264.7 cells (Jung et al. 2008). It also inhibited monosodium urate crystal-induced inflammation (Evan et al. 2008).

#### Resveratrol

Resveratrol, a natural active compound present in *Polygonum cuspidatum* has been reported to show promising curative effects on RA symptoms of CIA rats (Peng et al. 2013). It can induce the primary cultured FLS (rFLS) from CIA rats via up-regulation of Caspase-8 and down-regulation of FLICE inhibitory protein (FLIP). This inhibitory protein is a vital anti-apoptotic protein, which can suppress the death receptors ligands (such as TNF-α, FasL, and TRAIL) induced apoptosis in FLS of RA patients (RA-FLS) (Gu and Jin 2015).

#### Plant extract

Apart from single active compounds derived from plants, various studies have explored the effects of plant extracts in the treatment of RA. From the pharmacological perspective, natural plant extracts or mixed herbal compounds effectively regulate the immune system to alleviate RA by inhibiting pro-inflammatory cytokines (Zhao et al. 2021a, b).

In this context, Maresca and colleagues have evaluated the pharmacological activity of dried 50% hydroalcoholic extract (50%HA) of Astragalus (*Astragali radix*) using two different in vivo models of articular damage resembling RA and osteoarthritis. The authors demonstrated that administration of 300 mg/Kg per os of 50%HA significantly decreased both CFA-induced pain and monosodium iodoacetate (MIA)-induced pain (96% and 78% pain relief, respectively). In CFA model, 50%HA decreased the plasma level of pro-inflammatory cytokines IL-1β, TNF-α, and joint diameter (Maresca et al. 2017).

In a recent study, cinnamaldehyde (CA) in cinnamon (*Cinnamomum cassia Presland*) extract showed anti-inflammatory effects in in vitro experiments using activated macrophages (Raw246.7 cells) and in a rat model of adjuvant arthritis (AA) in vivo (Liu et al. 2020). Maresca and colleagues studied the acute effect of *Capparis spinosa* root extract on rat articular pain in osteoarthritis and RA rat models. Post 14th day CFA or MIA injection, different doses of *C. spinosa,* i.e., 300, 100, and 300 mg/kg of powdered roots, decoction, and hydroethanolic extract, respectively, were administered p.o. Significantly reduced hypersensitivity to noxious mechanical stimuli and spontaneous pain were evaluated as hind limb bearing alterations in both models. The methylene chloride (CH_2_Cl_2_) extract of *C. spinosa* and its correlated aqueous residue (30 mg/kg) were found to be the most effective in reducing the sensitivity to the pain (Maresca et al. 2016).

## Clinical studies on plant extracts and formulations containing plant extracts

In 1989, a placebo-controlled double-blind study was performed on an herbaceous plant of Southern China origin, *Tripterygium wilfordii,* involving 70 RA patients. A significant improvement was observed in the patients treated with the herbal drug compared to placebo after 3 months, in all the studied parameters like morning stiffness, swelling count, tenderness score, and grip strength at the end of treatment (Soeken et al. 2003).

In a randomized double-blind (RDB), single-center, placebo-controlled, parallel efficacy, drug trial in phase II, RA-1(a standardized formulation), composing purified plant extracts of *Zingiber officinale, B. serrata, C. longa,* and *W. somnifera,* was evaluated on 182 patients with active-on-chronic RA for 16 weeks. As a rescue analgesic, oral paracetamol and prednisolone (a fixed-dose not exceeding 7.5 mg daily) were permitted. This study was significant in terms of (i) enhanced patient percentage with a 50% decrease in the swollen joint count and swollen joint score, (ii) improved blood haemoglobin, (iii) a reduced RF titre. The ACR (American College of Rheumatology) 20% improvement index response was observed in 39% of the RA-1 group versus 30% of placebo. Only minor side-effects were reported in treatment groups with no report of drug toxicity.

A significant improvement in all ACR core efficacy variables and a modified version of HAQ were observed at weeks 32 and 54. Thus, this herbal formulation containing plant extracts was established as a good DMARD with a significant safety profile (Chopra et al. 2010).

A multicenter, RDB placebo-controlled phase III drug trial was conducted for 3 months on 130 patients, followed by a single-centre, open-label phase trial of 9-months, involving IRA-01 with extracts of *C. longa* (Turmeric), *B. serrata* (Salai Guggul), *Camellia sinensis* (Green tea), *Linum usitatissimum* (Flaxseed), *Trigonella foenum-graecum* (Fenugreek), *Piper nigrum* (Black pepper) and *Tribulus terrestris* (Gokshur). Prednisolone, DMARDs, or NSAIDs, were not allowed during this study period. Only paracetamol was qualified as a rescue analgesic. During the RDB phase, in comparison to the placebo, the above extract formulation showed better improvement in all efficacy measures, but achieved significance only in the case of physician global assessment of disease activity (Mann Whitney, *Z* = 2.18; 95% CI of change − 1.15, − 0.01). Only minor side-effects were recorded without significant adverse effects on metabolic parameters, biochemistry (renal and hepatic), or routine hematology during the entire study period. After 3 months, 58 patients went on to complete a 1-year follow-up, and 70 patients entered the open-label phase. A significant improvement in all efficacy variables, including joint pain and swelling, was observed. Here, 80% and 40% patients showed ACR 20 and 50 improvement response, respectively (Chopra et al. 2010).

In a study, 125 patients with joint pain were screened. Eighty-six patients satisfied inclusion criteria and took Ashwagandha powder (5 g, 2 times a day for 3 weeks) with lukewarm water or milk. SidhMakardhwaj (100 mg/day) with honey was given for successive 4 weeks. The patients were tested positive for increased ESR level and rheumatoid factor.

There was a significant reduction in RA factor after treatment with the above two herbal drugs along with the drastic change in scores of swollen joint counts, patient global assessment score, tender joint counts, patient self-assessed disability index score, physician global assessment score, pain assessment score ESR level in comparison to baseline.

In 56.4% of patients, ACR20 response and in 39.74% patients’ moderate response [European League against Rheumatism (EULAR) criteria] was observed. Normal kidney and liver function tests and increased urinary mercury levels were observed in the treated patients after 7 weeks (Kumar et al. 2015).

An investigator-blind, multicentre, parallel efficacy, three-arm (two Ayurvedic and Hydroxychloroquine sulfate (HCQS)) drug trial study was conducted on 121 patients for 24 weeks with active moderately severe RA (ACR 1988 classified) using polyherb (*Z. officinale* and *Tinospora cordifolia*) and monoherb (*Semecarpus anacardium*). The study measures included pain visual analog scale, joint counts (pain/tenderness and swelling), health assessment questionnaire, and global disease assessments. In the polyherb, monoherb, and HCQS arms, 44%, 36%, and 51%, respectively, improved ACR 20 index.

In the HCQS and polyherb groups, there was an improvement in efficacy measures no difference among the groups (significant P values). But the polyherb was observed better than monoherb. There were no differences between the groups with only mild adverse events (skin and gut, and none of them withdrew) were reported. Thus in this study, in comparison to HCQS, standardized Ayurvedic polyherb drug was found to be safe and effective in active RA patients (Chopra et al. 2012).

## Conclusion and future aspects

Rheumatoid arthritis manifests as an enduring, dynamic, and debilitating autoimmune condition, marked by persistent inflammation affecting bone joints and leading to damage in the surrounding ligaments. This disease extends its impact beyond the joints, affecting internal organs such as the lungs, heart, and eyes. Despite the prevalent use of synthetic drugs in the standard treatment of rheumatoid arthritis, the therapeutic journey is often marred by side effects. Regrettably, a definitive curative solution for rheumatoid arthritis remains elusive within contemporary medicine. While current medications effectively manage symptoms, offering relief from pain and joint inflammation, a true cure remains absent. Alternative treatments, including herbal remedies, various forms of massage therapy, gene therapy, yoga, and acupuncture, can be explored in conjunction with conventional approaches to alleviate pain and inflammation in the joints. However, the challenge persists in refining evaluation methodologies and introducing novel modalities to mitigate potential adverse reactions. The quest for more effective and targeted therapeutic interventions continues, seeking not only to manage symptoms but to address the root causes of rheumatoid arthritis for a more comprehensive and lasting relief.

## Data Availability

Enquiries about data availability should be directed to the authors.
